# In Vitro Propagation of Endemic Kazakh Tulips: Effects of Temperature and Growth Regulators

**DOI:** 10.3390/plants14193014

**Published:** 2025-09-29

**Authors:** Damelya Tagimanova, Olesya Raiser, Balsulu Kubentayeva, Gulden Nagmetova, Ainur Turzhanova, Oxana Khapilina

**Affiliations:** 1 National Centre for Biotechnology, Korgalzhin hwy 13/5, Nur-Sultan 010000, Kazakhstan; 2008olesya@mail.ru (O.R.); gulden30-04@mail.ru (G.N.); turzhanova-ainur@mail.ru (A.T.); 2 Astana Botanical Garden, Orynbor, 14e, Astana 010000, Kazakhstan; balsulu1992@mail.ru

**Keywords:** biodiversity, endemic species, growth regulators, in vitro culture, micropropagation, *Tulipa* sp.

## Abstract

*Tulipa auliekolica* and *Tulipa turgaica* have been recently described as endangered species endemic to Kazakhstan, which require urgent conservation amid rising human impact and climate change. Biotechnology offers effective tools for conserving such rare species; however, species-specific in vitro protocols tailored to their biological traits remain largely unreported. This study aimed to develop an in vitro propagation protocol for these rare *Tulipa* species by investigating the effects of different temperature regimes and phytohormone treatments. We conducted a study on the in vitro propagation of two recently described species, *T. auliekolica* and *T. turgaica*. Species-specific temperature regimes for seed stratification were established. Maximum germination of *T. auliekolica* was achieved at alternating temperatures of 4/10 °C, and of *T. turgaica* at 10/20 °C. No seed germination from either species occurred at a constant temperature of 20 °C. Bulbs cultured on Murashige & Skoog (MS) medium supplemented with 90 g/L sucrose and the growth regulators mT (meta-topolin) and BAP (6-benzylaminopurine) were effective in stimulating the formation of up to 4–7 microbulbs. Cultivation on a medium supplemented with 0.5 mg L^−1^ IBA (indole-3-butyric acid) resulted in the formation of mature bulbs covered with scales. These results can be successfully used in biodiversity conservation programs for the endemic *Tulipa* species. In addition, they provide a valuable basis for future biotechnological research, including microclonal propagation, the establishment of gene banks, and the development of reintroduction methods for Kazakh endemic *Tulipa* species.

## 1. Introduction

Kazakhstan is located at the heart of Eurasia and globally ranks ninth by land area, encompassing nearly all major natural landscape types found on Earth. The ecological and climatic diversity of this region fosters a rich and unique flora, characterised by a high level of endemism. The country’s vascular plant flora includes 451 endemic taxa, accounting for approximately 7.97% of Kazakhstan’s total floristic diversity [1].

Among the genera reflecting the region’s endemism, *Tulipa* is notable for its high species diversity and broad ecological adaptability across Kazakhstan [2,3]. This genus comprises 41 species in Kazakhstan, 13 of which are endemic [4]. Nine of these endemics are found in Northern and Central Kazakhstan, including *T. alberti* Regel and the recently described *T. turgaica* Perezhogin and *T. auliekolica* Perezhogin [5]. The restricted distribution and vulnerability of these species to environmental, anthropogenic, and climatic pressures—such as overgrazing and uncontrolled harvesting—necessitate the urgent need for research and targeted conservation measures to mitigate habitat degradation [6,7].

Risk assessment for endemic plant extinction in Kazakhstan is predominantly local in scope and lacks integration into global conservation frameworks. Therefore, many species face the threat of extinction without being adequately documented or protected [8,9]. In this context, studies on the conservation of endemic *Tulipa* spp. within steppe-type plant communities are of critical importance [9,10].

Various complementary strategies are employed to conserve plant biodiversity, including in situ conservation in natural habitats and ex situ methods outside native environments, such as botanical gardens, seed banks, cryogenic storage facilities, and in vitro collections. In situ approaches are often limited in effectiveness due to high costs, implementation complexity, and susceptibility to external disturbances. Consequently, biotechnological methods are increasingly used to support both the conservation and propagation of rare taxa [11].

Optimised in vitro culture systems are essential for conserving endemic species by enabling microclonal propagation, medium-term repositories, and cryo-preservation, thus supporting sustainable strategies for germplasm banking, population recovery, and restoration of ex situ collections [12]. Such techniques have been successfully applied to several rare and endemic species [13,14]. However, for recently described and endangered taxa, species-specific in vitro protocols—tailored to their biological characteristics and conservation—need to be developed and optimised.

Currently, there is no available information on the effects of temperature regimes and growth regulators on in vitro bulb formation in *T. turgaica* and *T. auliekolica*, which is primarily due to the lack of prior biotechnological studies involving these narrow-range endemic species. The combination of temperature and phytohormonal conditions plays a crucial role in regulating plant growth and morphogenesis, and in directing the accumulation and translocation of assimilates to storage organs [15]. However, no data are available on the effects of temperature regimes and growth regulators on in vitro bulb formation in *T. turgaica* and *T. auliekolica*, due to the lack of biotechnological studies on these narrow-range endemic species.

In this study, we aimed to investigate the effects of temperature regimes and the phytohormonal composition of the nutrient medium on seed germination and in vitro bulb formation, with the goal of developing effective microclonal propagation systems for the rare and endemic tulip species *T. turgaica* and *T. auliekolica*. The development of effective micropropagation protocols is necessary to create systems for preserving vulnerable and rare plants in in vitro collections, which serve as a germplasm bank and a reserve fund for replenishing living collections in ex situ conditions.

## 2. Results

### 2.1. Population Status of T. auliekolica and T. turgaica

A population of *T. turgaica* was identified in the Amangeldy district of the Kostanay region (N 66.026724°, E 50.486579°), inhabiting a flat area with loamy soils. Phytocenosis was represented by a feather grass–wormwood–meadow sweet community, dominated by *Festuca valesiaca* and *Artemisia schrenkiana*. The density of generative individuals was 10.3 ± 0.6 individuals per 1 m^2^. The *T. auliekolica* population was recorded in the Auliekol district of the Kostanay region (N 64.395259°, E 51.983026°), also on a flat plain with chestnut loamy soils. The vegetation was characterised as a wheatgrass community, dominated by *Elytrigia repens*. The density of generative individuals was substantially lower, at 2.0 ± 0.8 individuals per 1 m^2^ (Table S1).

The morphometry of the species is presented in Table S2. Morphometric analysis revealed significant interspecific differences. *T. turgaica* exhibited greater height (14.43 ± 2.96 cm) compared to *T. auliekolica* (10.83 ± 1.67 cm). Seeds of *T. auliekolica* were larger (5.5 × 4.5 mm) than those of *T. turgaica* (3.5 × 2.8 mm), with a more developed embryo. The capsule morphology also differed—capsules of *T. auliekolica* were broader, whereas those of *T. turgaica* were more elongated. Despite these differences, the seeds of both species were morphologically similar—flat, triangular, brown-yellow, and arranged horizontally in two rows within each locule. Ecologically, the species occupied distinct soil types. *T. turgaica* was found in heavy clay soils, whereas *T. auliekolica* preferred dry, lighter-textured soils. The average density of generative individuals was lowest for *T. auliekolica,* with approximately 2 plants/m^2^.

### 2.2. Introduction to In Vitro Culture

#### 2.2.1. Establishment of Sterile Seedlings from *T. auliekolica* and *T. turgaica* Seeds

**Figure 1:**
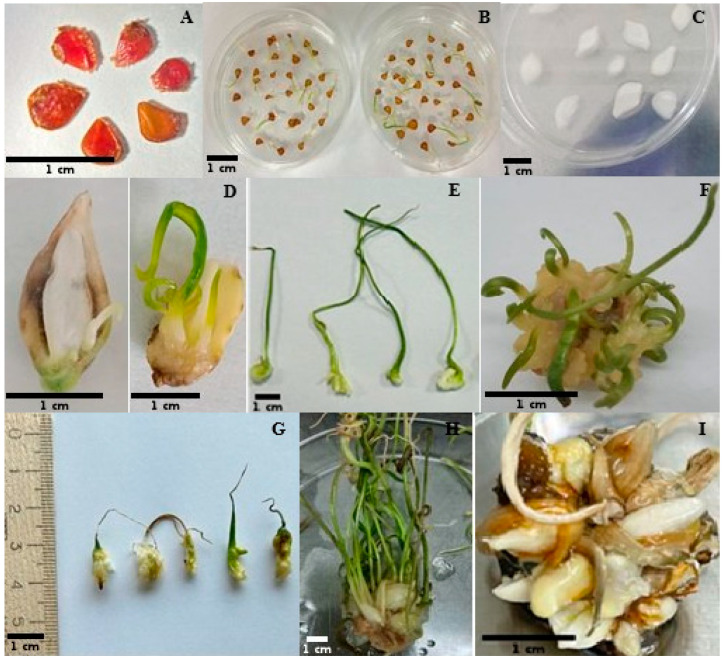
Introduction of *T. auliekolica* and *T. turgaica* explants into in vitro culture. (**A**) viability assessment of *Tulipa* spp. seeds; (**B**) germination of seeds; (**C**) cross-sections of *T. auliekolica* bulbs; (**D**) induction of microshoot development from bulb scale segments; (**E**) formation of microbulb primordia on seedlings; (**F**) development of *T. auliekolica* microshoots on MSP3 medium; (**G**) development of bulbs from seedlings on MSP3 medium; (**H**) bulb formation from microshoots on medium containing 90 g/L sucrose; (**I**) mature bulb development on MSB4 medium.

The evaluation of *T. auliecolica* and *T. turgaica* seeds using 2,3,5-triphenyltetrazolium chloride indicated high viability in the collection year. Prior to in vitro culture initiation, seed viability of *T. auliekolica* and *T. turgaica* was assessed using 2,3,5-triphenyltetrazolium chloride (TTC) staining. High viability was observed in seeds collected during the current year. *T. auliekolica* exhibited 100% viability, with all seeds staining a characteristic red, whereas *T. turgaica* showed 95% viability (Figure 1A). Non-viable seeds remained unstained, retaining their original coloration.

Analysis of *T. auliekolica* seed germination revealed statistically significant differences among temperature and medium conditions. Mean germination rates and results of Tukey’s post hoc test are summarised in Table 1.

At 20 °C, the average germination rate on all three media was zero, and no seeds germinated. A substantial increase in germination occurred at 10 °C and 10/20 °C, with rates rising to 48–57% across all media. At 10 °C, mean germination ranged from 48.0% on the control to 54.0% on ½MS + 10 mg L^−1^ GA_3_ medium. These values were statistically similar for media at this temperature, based on post hoc comparisons (*p* < 0.05). Germination was further enhanced at 10/20 °C, with the highest value (57.0%) observed on ½MS + 10 mg L^−1^ GA3 medium, which differed significantly from several lower-performing combinations (*p* < 0.05). The highest germination rate (88.0%) occurred at 4/10 °C on ½MS + 10 mg L^−1^ GA3 medium, which was significantly greater than all other treatments (*p* < 0.001). In contrast, at the same temperature, germination was significantly lower on the control and ½MS media (*p* < 0.01).

Analysis of variance (ANOVA) revealed that both temperature (Df = 4, F = 1454.35, *p* < 0.001) and medium type (Df = 2, F = 112.59, *p* < 0.001) had significant effects on *T. auliekolica* seed germination. The interaction between temperature and medium was also significant (Df = 8, F = 12.91, *p* < 0.001), indicating that the effect of the medium type varied depending on the temperature (Table S3).

The optimum stratification temperature differed for the *T. turgaica* seeds. The highest germination rates (65–69.3%) were observed on ½MS medium supplemented with 10 mg L^−1^ GA_3_ at 10–10/20 °C (Table 2).

The ANOVA results indicated that temperature (F (4, 30) = 322.21, *p* < 0.001), medium type (F (2, 30) = 298.75, *p* < 0.001), and their interaction (F (8, 30) = 53.33, *p* < 0.001) had a significant effect on *T. turgaica* seed germination (Table S4).

A two-way ANOVA revealed that both temperature and medium composition had a significant effect on the time to 50% germination (T_50_) (*p* < 0.001). Significant interactions were also observed between temperature and medium composition (*p* < 0.001), as well as between temperature and plant species (*p* < 0.01), suggesting that plant responses to temperature may be species-specific. However, the main effect of plant species, their interactions with medium (medium × species), and with both temperature and medium (temperature × medium × species) on T_50_ were not statistically significant (*p* > 0.05). These results indicate that there is no evidence for a consistent effect of plant species or for higher-order interactions among the tested factors (Table S5).

**Figure 2:**
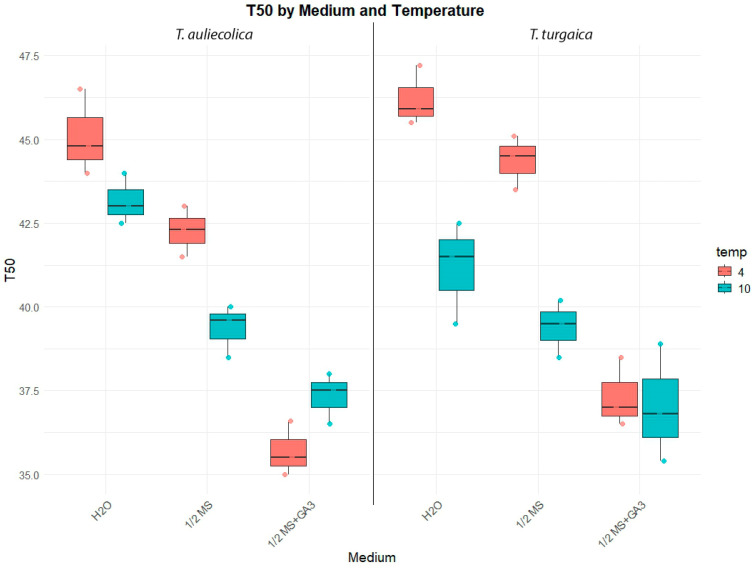
Time to 50% seed germination (T_50_, days) for *T. auliekolica and T. turgaica* seeds under different temperatures and media types. Boxplots show the distribution of T_50_ values at two temperatures (4 °C in red, 10 °C in blue) across three media: half-strength Murashige and Skoog (MS) medium (½MS), ½MS supplemented with 10 mg L^−1^ gibberellic acid (GA_3_) (½MS + GA_3_), and distilled water (H_2_O). The central line represents the median, boxes indicate the interquartile range, and whiskers show the range excluding outliers.

Average T_50_ comparisons revealed the longest germination times (45.1 days for *T. auliekolica* and 46.2 days for *T. turgaica*) under the control treatment (H_2_O) at 4 °C. The shortest T_50_ (37.03 days for *T. turgaica* at 10 °C and 37.33 days for *T. auliekolica* at 4 °C) was on ½MS medium supplemented with 10 mg L^−1^ GA_3_ (Figure 2).

Introducing GA_3_ to the culture medium significantly accelerated seed germination compared to the control. Optimal germination temperatures differed between the two endemic species. For *T. auliekolica*, the optimum was observed at 4 °C, with germination declining at temperatures above 10 °C. In contrast, *T. turgaica* exhibited optimal germination at 10 °C. Supplementation of the medium with GA_3_ at a concentration of 10 mg L^−1^ significantly increased both the germination rate and final germination percentage. These findings have practical implications for developing efficient in vitro propagation protocols for rare and endemic *Tulipa* species.

#### 2.2.2. Microshoot Induction from *T. auliekolica* and *T. turgaica* Bulbs

Microshoot development from fragmented bulb segments was first observed 20–30 days after culture initiation on the MS medium supplemented with 3 mg/L thidiazuron (TDZ) and 1 mg/L 1-naphthylacetic acid (NAA), primarily originating from the basal region of the explants. Initial morphological changes included an increase in scale size and the appearance of morphogenic foci on the inner surface, from which microshoots subsequently emerged. Up to 4–10 microshoots were noted per explant (Figure 1C–F).

Species-specific differences in the timing of microshoot formation rates were identified. The average time to shoot formation was significantly shorter in *T. auliekolica* (22.5 ± 1.5 days) compared to *T. turgaica* (32.6 ± 1.5 days; ANOVA, *p* < 0.001) (Table 3).

Significant interspecific differences were observed in the dynamics of shoot morphogenesis in vitro. Shoot formation frequency was also higher in *T. auliekolica* (95.7 ± 2.1%) than in *T. turgaica* (91.3 ± 2.0%; *p* < 0.001). Moreover, the mean number of shoots per explant was significantly greater in *T. auliekolica* (2.8 ± 0.3) than in *T. turgaica* (1.7 ± 0.3; *p* < 0.001).

### 2.3. Micropropagation

#### 2.3.1. Seedling Development from Seeds

The length of the microshoots derived from seeds was influenced more by medium composition than by species. *T. turgaica* seedlings exhibited shorter shoot lengths compared to *T. auliekolica* seedlings. The shortest shoots were recorded for the hormone-free MS control. In contrast, the most vigorous growth for both species was observed for seedlings cultured on MSP3 medium supplemented with 0.1 mg/L meta-topolin (mT), 0.1 mg/L Naphthaleneacetic Acid (NAA), and 5 mg/L 6-Benzylaminopurine (BAP). On this medium, the maximum shoot length recorded was 11.8 ± 1.5 cm for *T. auliekolica* and 8.8 ± 1.9 cm for *T. turgaica*. MSP3 medium proved to be the most effective across all evaluated parameters (Table 4). Multiple shoots or callus formation were not observed under any treatment for seedlings of either species. After 18 weeks of cultivation on MSP3 medium, microbulbs and primary roots were formed inside individual seedlings of both species (Figure 1G). Seedlings were maintained in this medium until mature microbulbs developed.

#### 2.3.2. Microshoot Development from Bulbs

The results of two-way ANOVA revealed statistically significant differences among the cultivation media in the number of explants for each species (Table 5). In *T. auliecolica*, the number of shoots ranged from 2.0 ± 0.7 in the control to 9.6 ± 3.3 on MSP3 medium, the latter being significantly higher than in all other treatments. Shoot length in this species was generally low (1.7–2.7 cm) across most variants, but markedly increased on MSP3 (7.7 ± 1.3 cm). The minimum indices for the number of shoots and their lengths were obtained for the control variant. The highest number of shoots—9.6 ± 3.3 pieces per explant in *T. auliecolica* species was obtained on the MSP3 medium variant.

The lowest values for shoot number and length were recorded for the hormone-free control. The highest shoot number for *T. auliekolica*—9.6 ± 3.3 shoots per explant—was observed on the MSP3 medium (0.1 mg/L mT, 0.1 mg/L NAA, and 5 mg/L BAP). The lowest number of shoots—2.5 ± 0.8 per explant—was observed on the MSP2 medium containing 0.1 mg/L TDZ, 0.1 mg/L IMS, and 5.0 mg/L BAP. In contrast, *T. turgaica* exhibited the highest values for all measured parameters on the MSP3 medium.

Each shoot was excised from the explant and subcultured three times onto fresh medium of similar composition, with each subculture lasting six weeks. The frequency of shoot formation significantly declined with each subsequent subculture, resulting in only 1–3 adventitious shoots per explant, depending on the medium composition. MSP3 and MSP5 media notably enhanced shoot proliferation. The average number of newly formed microshoots ranged from 3.21 to 3.4 for *T. auliekolica* and from 2.46 to 3.02 for *T. turgaica* (Table 6). By the end of the second subculture, microbulb formation was observed at the base of each microshoot. Analysis of various phytohormone combinations revealed that replacing the auxin indole-3-butyric acid (IBA) (culture media variants MSP2, MSP4 and MSP6) with NAA (culture media variants MSP1, MSP3 and MSP5) significantly reduced shoot formation.

The regenerated shoots were weak and did not survive. Results of a two-way ANOVA indicated that both subculture number (*p* < 0.001) and culture medium composition (*p* < 0.001) had statistically significant effects on shoot length in both *Tulipa* species. However, their interaction was not statistically significant (*p* > 0.05).

### 2.4. Microbulb Formation

**Figure 3:**
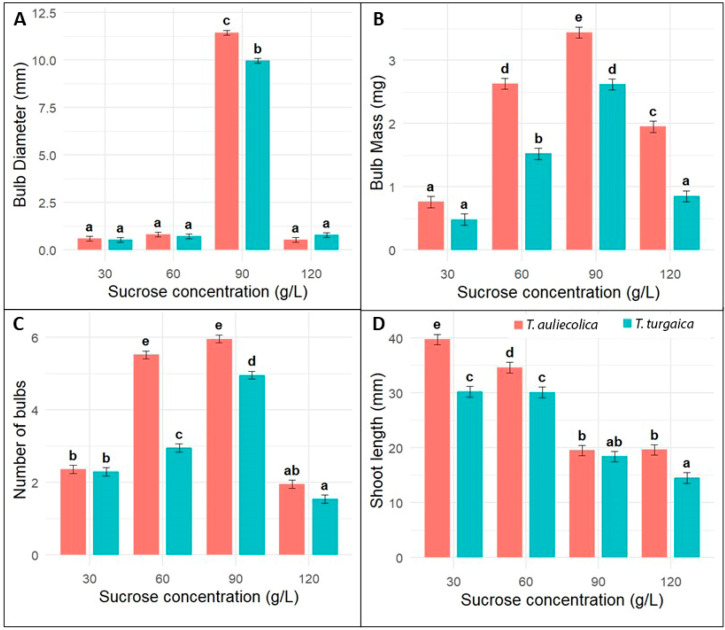
Effects of sucrose concentration on bulb formation from seedlings of *Tulipa auliekolica* and *T. turgaica* ((**A**)—Bulb diameter, (**B**)—Bulb mass, (**C**)—Number of bulbs, (**D**)—Shoot length).Bar charts represent mean ± standard error (SE) for bulb diameter (mm), bulb mass (mg), bulb number, and shoot length (mm) at different sucrose concentrations (g/L). Different letters indicate statistically significant differences among treatments within each species based on Tukey’s post hoc analysis (*p* < 0.05).

Microbulb formation was induced by culturing both types of explants—seedlings and microshoots—on the optimal MSP3 medium supplemented with varying sucrose concentrations. Seedling-derived cultures did not produce mature bulbs; only isolated microbulbs, 2–3 mm in diameter, were observed on single sprouts after 4–6 months of cultivation (Figure 3).

**Figure 4:**
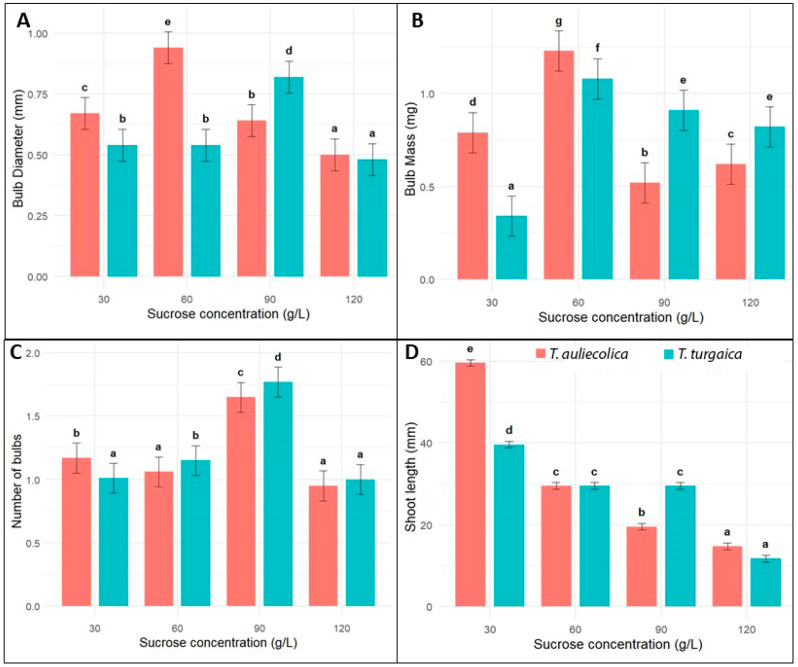
Effects of sucrose concentration on bulb formation from microshoots of *T. auliekolica* and *T. turgaica* ((**A**)—Bulb diameter, (**B**)—Bulb mass, (**C**)—Number of bulbs, (**D**)—Shoot length). Bar graphs represent mean ± standard error (SE) for bulb diameter (mm), bulb mass (mg), bulb number, and shoot length (mm) across different sucrose concentrations (g/L). Different letters above bars indicate statistically significant differences (*p* < 0.05) within each species and parameter, based on post hoc analysis.

In contrast, microshoot-derived explants exhibited active and consistent microbulb formation, which was strongly influenced by the sucrose concentration in the MSP3 medium. The initial stages of bulb formation were characterised by halted leaf growth, swelling at the shoot base, and progressive yellowing—morphological indicators of bulb initiation. Results of one-way ANOVA revealed that sucrose concentration had a statistically significant effect on all measured parameters for both *Tulipa* species (*p* < 0.05; Figure 4).

For *T. auliekolica* and *T. turgaica* microshoots, the microbulb formation was strongly dependent on the sucrose concentration in the nutrient medium. The highest numbers of microbulbs—5.96 ± 0.4 for *T. auliekolica* and 4.96 ± 0.5 for *T. turgaica*—were achieved at a sucrose concentration of 90 g/L. This concentration also yielded the largest bulb diameter and weight, with values significantly higher than those at other concentrations. No significant differences were observed among the other sucrose concentrations, except at 120 g/L, where a marked reduction in microbulb formation occurred. At this high sucrose level, only 1.95 ± 0.2 microbulbs for *T. auliekolica* and 1.54 ± 0.3 for *T. turgaica* were formed, with the microbulbs being notably smaller and lighter. This reduction was possibly due to the inhibitory effect of excess sugar on growth and biomass accumulation. Although microbulb formation was also observed at 60 g/L sucrose concentration, the bulbs produced were significantly smaller and lighter than those formed at 90 g/L (Figure 1H). These results confirmed that the optimal sucrose concentration for maximum microbulb growth was 90 g/L, and that both lower and higher concentrations negatively affect development.

To promote the formation of mature bulbs—characterised by the presence of protective scales—microbulbs were further cultured on media supplemented with various auxins. Six different nutrient media were evaluated at the bulb formation stage, each producing distinct results in microbulb number per explant and bulb weight. Statistically significant differences were observed across all treatments (*p* < 0.05), and the Sidak post hoc test identified the specific groups with significant variation (Table 7).

The highest number of microbulbs for *T. auliekolica* (7.69 ± 1.4) and *T. turgaica* (6.18 ± 0.6) was recorded on the MSB4 medium, significantly exceeding all other treatments. The medium also produced the heaviest microbulb for both species. In contrast, the lowest microbulb numbers were observed on MSB-3: 3.46 ± 0.8 for *T. auliekolica* and 1.98 ± 0.7 for *T. turgaica*. Media MSB1, MSB2, and MSB5 yielded intermediate microbulb weights ranging from 1.06 ± 0.4 mg to 1.52 ± 0.3 mg, with partial statistical overlap between groups (Figure 1I). Overall, MSB4 was the most effective medium for microbulb production and weight for both tulip species, whereas MSB3 exhibited the poorest performance.

## 3. Discussion

The preservation of biological diversity is a central objective in modern biology, particularly in the face of increasing anthropogenic pressures. Endemic species with restricted distribution and small population sizes are particularly vulnerable. Kazakhstan, as part of the Central Asian genetic centre, is a critical region for biodiversity conservation. Despite the ecological importance of endemic plants in Kazakhstan, their extinction risk is often assessed at a local rather than global scale. Consequently, many species may disappear unnoticed [8,9]. Research on newly identified endemic *Tulipa* species, particularly those found in steppe-type plant communities, is therefore of high priority [9,10].

Recent advancements in plant conservation have introduced effective biotechnological approaches. In vitro methods, such as microclonal propagation, cryopreservation, and callus cultures, enable the preservation of genetic resources in rare and endangered species. Although tulips are generally challenging to propagate in vitro, successful protocols have been established for several species using direct organogenesis from seedling segments and microbulb scales [16,17,18,19]. However, to date, no such studies have been conducted on *T. turgaica* and *T. auliekolica*. Considering the ecological and morphological differences observed between these endemic species, optimising each stage of micropropagation is essential, with particular focus on determining optimal germination temperatures and assessing the effects of exogenous growth regulators on in vitro morphogenesis. *T. turgaica* grows on heavier, loamy soils and forms dense populations with approximately 10 specimens/m^2^, suggesting more favourable habitat conditions. In contrast, *T. auliekolica* prefers dry, light soils and occurs at much lower densities (2 individuals/m^2^), highlighting extreme vulnerability and the urgent need for conservation efforts.

Morphometric analysis confirmed clear species-level differences. *T. turgaica* plants were taller and produced more elongated capsules, whereas *T. auliekolica* was characterised by larger seeds with well-developed embryos and more rounded capsules. Such morphological traits reflect adaptation to distinct ecological niches and are critical for optimising in vitro micropropagation protocols and conservation strategies for rare and endemic species. Temperature sensitivity during seed germination—a strictly deterministic trait common among geophytes, including tulips—can widely vary even within a single plant family [20].

In the present study, notable differences in optimal germination temperature were observed between the two species. Although seeds of both species started germination on Day 35, their responses to stratification differed. *T. auliekolica* seeds achieved their highest germination rate (88%) under a 4/10 °C stratification regime, indicating a strong requirement for low temperatures. In contrast, *T. turgaica* seeds showed optimal germination (69%) at 10/20 °C, suggesting a lesser dependence on cold stratification. These findings are consistent with the species’ natural habitats: *T. auliekolica* occupies drier, lighter soils and is ecologically adapted to low-temperature germination, whereas *T. turgaica*, which grows in heavier loamy soils, requires warmer conditions to break dormancy [4]. Notably, both *Tulipa* species showed an absence of seed germination at a constant temperature of 20 °C. A similar temperature-dependent germination pattern has been reported for other Central Asia members of the *Alliaceae*. Temperature thresholds for germination in *Allium* are species-specific, and even a slight increase in temperature can markedly reduce both the rate and efficiency of germination [21]. A study on Greek endemic tulip species demonstrated that seeds germinated at 5 °C, and no germination was observed at 20 °C [17,19]. This suggests the presence of a thermoinhibition mechanism—a trait commonly found in plant species adapted to sharp continental climates [17,22,23]. Thermoinhibition serves an important ecological role by preventing seed germination under suboptimal conditions and ensuring synchronisation of the plant life cycle with seasonal climatic patterns [24].

However, this adaptive mechanism is threatened by global climate change. According to the latest Intergovernmental Panel on Climate Change projections, the average annual temperature in Central Asia could rise by 2–3 °C by 2050 [25]. Such changes threaten highly specialised endemic species with low ecological plasticity, limiting their capacity to rapidly adapt or expand their ecological niches in response to shifting environmental conditions. In most *Tulipa* species, the morphological immaturity of the embryo necessitates further development under low-temperature conditions (cold stratification). At the same time, the physiological component of dormancy is largely controlled by phytohormones, primarily abscisic acid (ABA) and gibberellins (GA) [20]. Prolonged exposure to elevated temperatures during dormancy disrupts this balance by enhancing ABA accumulation and reducing GA activity, thereby extending the dormancy period [26]. Under ongoing climate warming, the shortening of cold seasons interferes with both the required duration of low-temperature exposure and the associated hormonal restructuring in seeds. As a result, germination may be strongly suppressed in endemic *Tulipa* species with a pronounced requirement for stratification under reduced temperatures [24]. Such deviations from climatic norms heighten the risk of impaired embryogenesis and hinder the development of viable embryos capable of establishing a new sporophyte generation [19]. In addition to temperature, the application of GA_3_ at 10 mg L^−1^ significantly enhanced seed germination. This finding supports the role of GA_3_ in overcoming seed dormancy in tulips and other geophytes [26,27,28]. GA_3_ is particularly effective in alleviating physiological dormancy—a characteristic feature of many bulbous plants. Its mode of action involves stimulating hydrolytic enzyme activity, reducing the levels of inhibitory hormones such as abscisic acid (ABA), and improving overall seed physiological status [29,30]. In the present study, despite the promotive effects of GA_3_ on seed germination, no germination was observed when tulip seeds were cultured at a constant temperature of 20 °C. This finding suggests a temperature-dependent response to GA_3_, possibly mediated by mechanisms that reduce tissue sensitivity to this phytohormone at elevated temperatures [24,31]. High temperatures activate the ABA biosynthesis by upregulating the expression of genes such as *ZEP*, *NCED2*, *NCED5*, and *NCED9*, leading to an increased ABA accumulation. ABA suppresses germination and maintains physiological dormancy. Concurrently, elevated temperatures may inhibit gibberellin biosynthesis, which exacerbates seed dormancy, due to the essential role of GAs in dormancy release and the initiation of embryo growth [32]. Thus, high temperatures disrupt the hormonal balance required for germination, characterised by elevated ABA levels and reduced GA levels, jointly inhibiting seed germination.

It is therefore plausible that tulip seeds exhibit a threshold temperature above which the GA_3_-mediated signalling cascade is impaired, or cellular sensitivity to GA_3_ is reduced. Considering the temperature sensitivity and species-specific responses, effective in vitro germination protocols should optimise both GA_3_ concentration and incubation temperature regime for each *Tulipa* species. Only under such optimised conditions can physiological dormancy be reliably overcome, enabling the production of viable seedlings for subsequent micropropagation and conservation of rare *Tulipa* species.

In the present study, we used transverse sections of fragmented bulb tissues from two tulip species—*T. auliekolica* and *T. turgaica*—to obtain microshoots. Morphogenesis in both species started with the enlargement of scale tissues and the emergence of morphogenic points on their inner surfaces when cultured on MS medium supplemented with 3 mg/L TDZ and 1 mg/L NAA. A similar morphogenetic pattern has been observed in other *Tulipa* species, where preferential bud formation occurred in the basal part of the explants [33], possibly due to the high meristematic activity of the basal meristem typical of bulbous plants [34,35,36,37]. In the present study, significant interspecific differences were observed in the rate and efficiency of shoot formation. *T. auliekolica* explants initiated microshoots more rapidly (20–25 days) compared to *T. turgaica* (30–35 days). Comparable interspecific variation in morphogenesis timing has been reported in other *Tulipa* species, possibly reflecting genetic factors and adaptations to their native ecological or geographical conditions [38]. Both species exhibited high shoot induction frequencies—95% for *T. auliekolica* and 92% for *T. turgaica*. However, the average number of microshoots per explant differed—*T. auliekolica* produced 2.8 shoots per explant, whereas *T. turgaica* formed only 1.6. These differences in regenerative capacity may result from species-specific differences in endogenous hormonal status [39,40,41]. The combination of TDZ (a cytokinin) and NAA (an auxin) used in the present study proved effective in inducing morphogenesis in both species, consistent with previous studies demonstrating the high efficiency of TDZ in the micropropagation of various plant taxa [42,43,44,45]. TDZ is a diphenylurea derivative with cytokinin-like activity that stimulates cell division and bud initiation in the nodal segments of plants, resulting in rapid shoot formation through enhanced cytokinin signalling. The high efficacy of TDZ in promoting shoot proliferation is attributed to both direct interaction with cytokinin receptors [46] and the indirect enhancement of endogenous cytokinin levels via suppression of cytokinin oxidase/dehydrogenase activity. TDZ often outperforms conventional cytokinins such as BAP or kinetin in stimulating shoot proliferation [47,48,49]. However, prolonged exposure to TDZ can lead to morphological and physiological abnormalities, including hyperhydricity and genomic instability. Therefore, in subsequent stages of micropropagation, we replaced it with alternative cytokinins such as mT, BAP, or (2iP) to minimise these adverse effects [50].

To induce microbulb formation, seedlings and microshoots were subcultured onto MS medium supplemented with 30 mg/L sucrose, 5 mg/L BAP, and various growth regulators. The MSP3 medium proved most effective, promoting maximum microshoot elongation from seed-derived material—reaching up to 12 cm in *T. auliekolica* and up to 9 cm in *T. turgaica*. Despite the formation of only one shoot per seedling, rudimentary microbulbs were observed at the basal part of the shoot after 18 weeks of cultivation on MSP3, indicating the medium’s potential for initiating the microbulb formation stage in micropropagation.

In bulb-derived microshoot cultures, shoot induction and elongation were also influenced by the composition of the medium. *T. auliekolica* explants exhibited higher regenerative capacity, producing up to ten shoots per explant, compared to *T. turgaica*, which formed a maximum of four. The lowest shoot induction rates and shoot lengths were recorded on MSP2 medium containing TDZ, supporting prior observations regarding the adverse effects of prolonged TDZ exposure on tulip physiology and metabolism.

The intensity of de novo shoot formation was maximal during the first subculture on MSP3 medium, with *T. auliekolica* and *T. turgaica* producing up to 4.5 and 4.0 shoots per explant, respectively. However, shoot regeneration declined in subsequent subcultures. This reduction may be attributed to a physiological shift in the allocation of metabolic resources towards microbulb development, leading to decreased meristematic activity and limited shoot growth. Similar trends were reported in other studies, where the combined application of cytokines and auxins promoted both shoot elongation and microbulb induction [51]. Comparison of different media variants revealed that replacing NAA with IBA in combination with TDZ and BAP reduced the efficiency of micropropagation. The MSP2 medium exhibited the lowest productivity, possibly due to the excessive cytokinin activity of TDZ, which can inhibit normal shoot development. In contrast, the MSP3 medium—containing mT and BAP—was the most effective, promoting both active shoot proliferation and microbulb initiation. A key factor contributing to the high efficiency of the MSP3 medium was the substitution of TDZ with mT. When combined with BAP and NAA, this formulation exhibited a pronounced synergistic effect, significantly increasing numbers and supporting the formation of microbulb primordia [52,53]. mT, an aromatic cytokinin derivative, differs from conventional BAP by exerting milder effects on plant tissues, promoting the development of physiologically stable shoots with a reduced incidence of hyperhydration and vitrification [54,55]. Its combination with BAP enhances the meristematic cell proliferation, while maintaining morphogenetic stability and facilitating timely differentiation [56].

To induce microbulb formation, seedlings and microshoots were further cultured on media containing varying sucrose concentrations. Sucrose serves not only as the primary carbon source in in vitro cultures but also as an osmotic regulator and signalling molecule, influencing key morphogenetic processes [57,58]. The present study highlights the pivotal role of sucrose concentration in regulating mature bulb formation in the studied tulip species, with both species exhibiting similar trends in morphometric parameters in response to varying sucrose concentration. Optimal values for microbulb number, diameter, and weight were observed at 90 g/L sucrose. In contrast, lower concentrations (30–60 g/L) and particularly high levels (120 g/L) significantly reduced these parameters. Elevated sucrose concentrations activate metabolite biosynthesis under in vitro conditions, reflecting a typical plant stress response, which suggests a potential inhibition of plant growth at high sucrose levels [15]. The observed reduction in shoot length at higher sucrose concentrations—particularly in *T. turgaica* (14.53 ± 2.8 mm at 120 g/L vs. 30.22 ± 4.3 mm at 30 g/L)—supports the inhibitory effect of increased osmotic potentials.

The negative correlation between shoot length and sucrose concentration aligns with the concept of carbohydrate balance, according to which increased osmotic pressure in the medium limits shoot growth, redirecting resources toward the formation of storage organs [59]. These findings confirm that sucrose exerts a multifactorial influence on the growth and development of tulip in vitro, functioning not only as a metabolic substrate but also as a regulator of physiological processes.

Additionally, the application of exogenous auxins influenced mature bulb formation. The highest number of microbulbs in both species was achieved using MSB4 medium supplemented with 0.5 mg/L IBA. Media supplemented with IAA showed a lower efficacy than those containing IBA, possibly due to the greater chemical stability of IBA in culture media and a slower metabolism in plant tissues [60,61,62]. The observed reduction in microbulb yield and weight at higher auxin concentrations, particularly in MSB3 and MSB6 media, may result from hormonal inhibition, where excessive levels of growth regulators suppress morphogenetic processes [63,64,65]. These findings have practical implications for the biotechnological propagation of rare endemic *Tulipa* species. Among the tested media, MSB4 supplemented with 0.5 mg/L IBA is recommended as an optimal medium for inducing microbulbs with high mass and yield.

Given the strong temperature sensitivity and species-specific responses, the application of GA_3_ in in vitro germination protocols must be coupled with an optimized temperature regime tailored to each *Tulipa* species. The success of ex situ propagation and subsequent reintroduction into natural habitats depends on accurately accounting for the species’ temperature requirements, stratification needs, and dormancy characteristics. Understanding these physiological traits is essential for designing effective storage and germination protocols. Moreover, this knowledge is critical for adapting conservation strategies to the realities of climate change, thereby ensuring the long-term sustainability of rare and vulnerable endemic *Tulipa* species.

## 4. Materials and Methods

### 4.1. Plant Material

**Figure 5:**
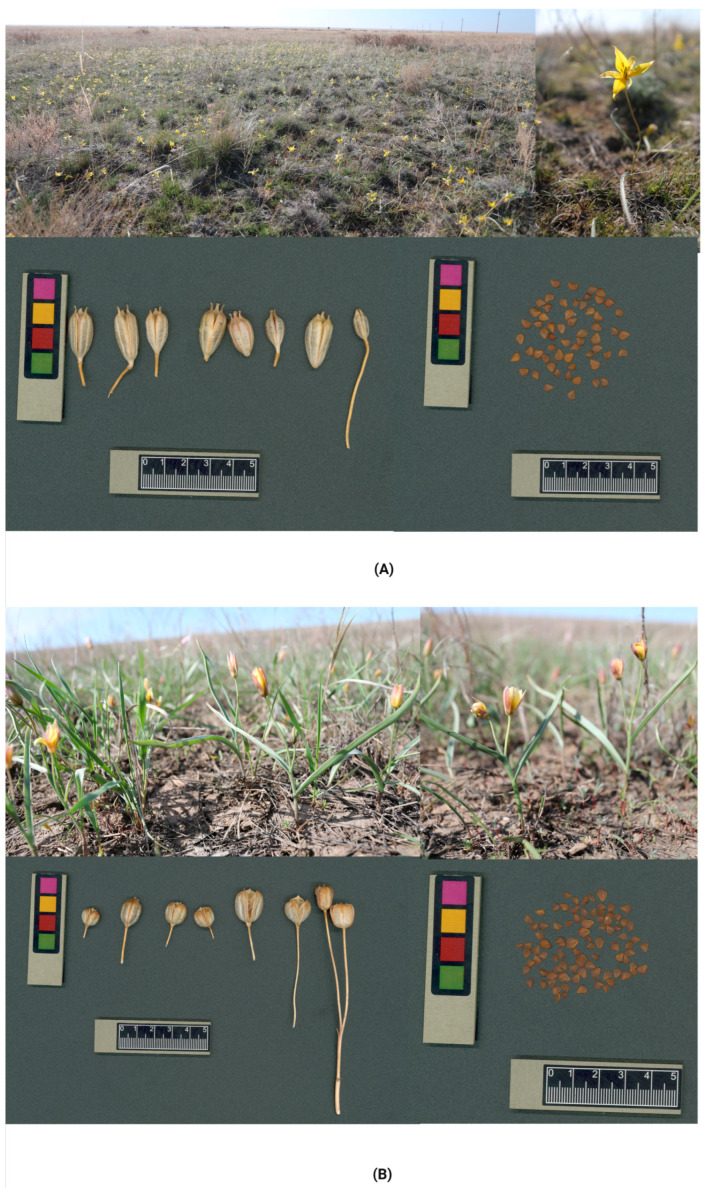
*Tulipa* sp. in their natural habitat in Kazakhstan. (**A**) *Tulipa turgaica* (**B**) *T. auliekolica* (Photos by S. Kubentayev).

Plant material included bulbs and seeds of the endemic tulip species *T. turgaica* and *T. auliekolica*, collected from natural habitats in Kazakhstan from 2023 to 2024. The species were identified using a special identification key from a botanical database [4,66]. The material was collected using a route-based sampling method combined with traditional morphological geographical approaches. Bulbs were harvested in April 2024 during the active vegetative phase (budding to flowering), whereas mature seeds were collected in August following fruiting (Figure 5). Healthy donor plants showing no visible signs of viral or fungal infection were selected from across the population range. Ten representative specimens were chosen per species. Bulbs were carefully excavated with soil residues intact, placed in containers and transported to the laboratory for further experimentation.

Voucher specimens were deposited in the herbarium of the Astana Botanical Garden under accession numbers NUR 0006542–NUR0006549. Molecular identification was further confirmed via DNA barcoding of the *mat K* gene regions. Sequences were submitted to GenBank at the National Centre for Biotechnology Information under accession numbers OR906694 and OR906696.

### 4.2. Tulip Plant Morphology

The morphological characteristics of *T. auliekolica* and *T. turgaica* were assessed using 10 parameters, with fifteen replicates per species. The evaluated traits included plant height, stem diameter, leaf blade length and width, number of flowers per plant, flower diameter, capsule length, bulb diameter, seed size, and seed weight. Seed size was measured in triplicate, each consisting of 100 seeds.

### 4.3. Determination of Seed Viability

Seed viability was assessed using a 10 g/L solution of 2,3,5-triphenyltetrazolium chloride (TTC). For each species, 20 seeds were tested in triplicate. Seeds exhibiting complete red staining were considered viable, whereas those with at least two-thirds staining of the basal portion of the seed coat were classified as conditionally viable. Viability was calculated as the percentage of viable seeds relative to the total number assessed.

### 4.4. Seed and Bulb Stratification Under In Vitro Conditions

Prior to stratification, bulbs and seeds were surface sterilised according to a previously established protocol involving treatment with 70% ethanol and 0.01% HgCl_2_ for 20 min [67]. Seed stratification was carried out under various temperature regimes: constant temperatures of 4 °C, 10 °C, and 20 °C, as well as alternating temperatures of 4/10 °C and 10/20 °C. The two-stage stratification involved sequential incubation for 4 weeks at each of the two different temperatures. Prior to in vitro culture, the bulbs were stored in the dark at 5 ± 2 °C for 4 weeks to break dormancy, a characteristic physiological requirement of all geophytes [19].

### 4.5. Seed Germination Under In Vitro Conditions

Sterilised viable seeds were cultured on half-strength MS (Sigma-Aldrich, St. Louis, MO, USA, M5519) supplemented with 10 mg L^−1^ GA_3_ (PhytoTech Labs, Lenexa, KS, USA, G500). Control treatments included seeds placed on ½MS medium without GA_3_ and on moist filter paper with distilled water. For each treatment, 20 seeds were used in triplicate. Seeds were considered germinated upon the emergence of a seedling petiole 1–2 mm in length. Germination was monitored daily for 60 days until no further germination occurred. The germination rate (%) was calculated as follows:

Germination percentage = (Number of germinated seeds/Total number of seeds) × 100%(1)

The time to 50% seed germination (T_50_) was calculated using the following equation [68]:

T50 = ti + ((N + 1)/2 − ni)/nj-ni)/(tj − ti)(2)

where N denotes the final number of germinated seeds, and ni and nj (ni < N/2 < nj) denote the total number of seeds germinated on the first (ti) and last (tj) days of cultivation, respectively.

### 4.6. Establishment of Aseptic Culture

Sterile bulbs were transversely sectioned into 1–2 mm thick slices and cultured on MS medium (Sigma-Aldrich, M5519) supplemented with 0.5 mg/L TDZ (Sigma-Aldrich, P6186) and 1.0 mg/L NAA (Sigma-Aldrich, N1641). Phytagel (Sigma-Aldrich, P8169) at 2 g L^−1^ was used as the gelling agent. All tissue culture media were adjusted to pH 5.6 with 1 N KOH, autoclaved at 121 °C for 20 min. PGRs were filter-sterilized by passing through 0.2 µm Millipore filters and added after autoclaving. Explants were incubated in the dark at 25–26 °C for 7–8 weeks.

To promote shoot proliferation from bulbs, various growth regulators were used, namely, auxins: NAA (Sigma-Aldrich, N1641) and IBA (Sigma-Aldrich, I5386); and cytokinins: TDZ (Sigma-Aldrich, P6186), 2iP (Sigma-Aldrich, D7660), mT (PhytoTech Labs, T841), and BAP (PhytoTech Labs, B800) (Table 8).

The concentration of BAP was maintained at 5.0 mg/L across all treatments. MS medium without growth regulators served as the control. Microshoots formed on bulb fragments were excised from the explants and transferred to a fresh medium of identical composition every 6 weeks for three successive subcultures. After the final subculture, the number and length of shoots, as well as the propagation rate, were measured. Seedlings derived from the seeds were cultivated under identical conditions.

All cultures were maintained in a BIOBASE^®^ (Jinan, China) climate incubator (BJPX-A1500CI) at 23 ± 2 °C, with a 16 h light/8 h dark photoperiod and an illumination intensity of 5000 lx.

### 4.7. Microbulb Induction and Development

Microshoot clusters derived from bulb fragments were separated using the same procedure applied to seed-derived seedlings and transferred to MS medium supplemented with varying concentrations of sucrose (30, 60, or 90 g/L) to induce microbulb formation. After 8 weeks, shoot growth, frequency of microbulb formation, and shoot biomass were assessed. The evaluated parameters included shoot length, number of microbulbs, bulb diameter, and fresh weight. After determining the optimal sucrose concentration, plants were subcultured on MS media supplemented with different auxins (IAA and IBA) at various concentrations to promote mature bulb development. The culture media differed in the type and concentration of auxins used (IAA and IBA), each of which was applied separately at concentrations of 0.5, 1, and 2 mg L^−1^. These cultures were maintained in the dark for 3 months without further subculturing.

### 4.8. Statistical Analysis

Data were analysed using ANOVA, followed by post hoc comparisons of mean values using Tukey’s HSD and Sidak tests. Results were reported using Compact Letter Display groups. All statistical analyses and visualisations were performed using the R Statistical Software (v4.2.3; R Core Team, 2023) [69].

## 5. Conclusions

The study demonstrated species-specific temperature sensitivity of seed germination in endemic species. *T. auliekolica* exhibited optimal germination under low-temperature stratification (4/10 °C), whereas *T. turgaica* required higher temperatures (10/20 °C). Constant exposure to 20 °C inhibited germination, possibly due to thermodormancy and hormonal imbalance. Adding gibberellic acid improves germination, but its effect is temperature-dependent and requires optimisation of the in vitro stratification conditions. The combined application of cytokinins and auxins (mT + BAP + NAA) resulted in the highest proliferative activity and significantly stimulated microbulb formation. A sucrose concentration of 90 g/L was optimal for bulb induction, with both lower and higher concentrations reducing the efficiency of mature bulb development. Among the tested auxins, IBA—at 0.5–2 mg/L—was most effective in inducing mature bulbs with protective cover scale formation. The results obtained from the optimisation of stratification regimes and microclonal propagation methods significantly enhanced the efficiency of the micropropagation of endemic tulips. These findings provide a foundation for the future development of in vitro genetic banks for rare and endemic plant species, and represent a promising approach for biodiversity conservation.

## Figures and Tables

**Table 1:** Seed germination rates of *T. auliekolica* under different temperature regimes and media types.

Seed Germination Rate (%)			
Temperature Regime (°C)	Media Type		
	Distilled Water (Control)	½MS	½MS + 10 mg L^−1^ GA_3_
4	58.0 ± 0.02 ^cd^	63.0 ± 0.02 ^de^	72.0 ± 0.01 ^f^
10	48.0 ± 0.03 ^b^	51.0 ± 0.02 ^b^	54.0 ± 0.01 ^bc^
20	0.0 ± 0 ^a^	0.0 ± 0 ^a^	5.0 ± 0.01 ^a^
4/10	62.0 ± 0.02 ^de^	68.0 ± 0.02 ^ef^	88.0 ± 0.02 ^g^
10/20	50.0 ± 0.02 ^b^	52.0 ± 0.02 ^bc^	57.0 ± 0.02 ^cd^

Note: ½MS, half-strength Murashige and Skoog medium; ½MS + 10 mg L^−1^ GA3, ½MS supplemented with 10 mg L^−1^ gibberellic acid. Temperature conditions indicate constant (4 °C, 10 °C, 20 °C) and alternating (4/10 °C, 10/20 °C) regimes. Values are presented as mean ± standard error. Different superscript letters within each column indicate statistically significant differences among treatments based on the Sidak post hoc test (*p* < 0.05).

**Table 2:** Mean germination rates (%) of *T. turgaica* under different temperature regimes and media types.

Seed Germination Rate (%)			
Temperature (°C)	Media Type		
	Distilled Water (Control)	½MS	½MS + 10 mg L^−1^ GA_3_
4	12.0 ± 0.02 ^b^	15.0 ± 0.03 ^b^	30.0 ± 0.02 ^c^
10	15.0 ± 0.03 ^b^	35.0 ± 0.04 ^cd^	65.0 ± 0.04 ^e^
20	0 ± 0 ^a^	0 ± 0 ^a^	0 ± 0 ^a^
4/10	20.0 ± 0.02 ^b^	20.0 ± 0.02 ^b^	35.0 ± 0.03 ^cd^
10/20	20.0 ± 0.02 ^b^	40.0 ± 0.02 ^d^	69.0 ± 0.01 ^e^

Note: ½MS, half-strength Murashige and Skoog medium; ½MS + 10 mg L^−1^ GA3, ½MS supplemented with 10 mg L^−1^ gibberellic acid. Temperature conditions indicate constant (4 °C, 10 °C, 20 °C) and alternating (4/10 °C, 10/20 °C) regimes. Values are presented as mean ± standard error. Different superscript letters within each column indicate statistically significant differences among treatments based on the Sidak post hoc test (*p* < 0.05).

**Table 3:** Comparison of microshoot formation in *T. auliekolica* and *T. turgaica*.

Species	Number of Explants	Days to Shoot Formation	Shoot Formation Frequency (%)	Number of Shoots per Explant
*T. auliekolica*	200	22.5 ± 1.5	95.7 ± 2.1	2.8 ± 0.3
*T. turgaica*	230	32.6 ± 1.5	91.3 ± 2.0	1.7 ± 0.3
*p*-value		<0.001	<0.001	<0.001

Note: Values are presented as mean ± standard error.

**Table 4:** Comparison of seedling lengths in *T. turgaica* and *T. auliekolica* across different culture media.

Culture Medium	Length of Shoots (cm)	
	*T. auliekolica*	*T. turgaica*
MS (control)	2.6 ± 0.8 ^a^	1.6 ± 0.7 ^a^
MSP1	3.5 ± 0.8 ^ab^	3.2 ± 1.2 ^ab^
MSP2	4.7 ± 1.5 ^bc^	3.9 ± 1.0 ^b^
MSP3	11.8 ± 1.5 ^d^	8.8 ± 1.9 ^c^
MSP4	5.6 ± 1.0 ^c^	4.5 ±1.4 ^b^
MSP5	3.5 ± 0.12 ^ab^	3.7 ± 1.2 ^b^
MSP6	4.5 ± 1.4 ^bc^	4.8 ± 1.3 ^b^

Note: MS, Murashige and Skoog medium. Values are presented as mean ± standard error. Different lowercase letters within each column indicate statistically significant differences among treatments based on Tukey’s post hoc test (*p* < 0.05).

**Table 5:** Effect of culture media on the number and length of shoots produced by bulb explants of *T. turgaica* and *T. auliekolica*.

Culture Medium	*T. auliekolica*		*T. turgaica*	
	Number of Shoots/Explant	Shoot Length (cm)	Number of Shoots/Explant	Shoot Length (cm)
MS (control)	2.0 ± 0.7 ^a^	1.7 ± 0.8 ^a^	1.3 ± 0.7 ^a^	1.6 ± 0.7 ^ab^
MSP1	5.0 ± 1.1 ^c^	2.4 ± 1.2 ^c^	3.5 ± 1.2 ^bc^	2.1 ± 0.7 ^ab^
MSP2	2.5 ± 0.8 ^ab^	1.8 ± 0.8 ^ab^	2.2 ± 0.8 ^ab^	1.3 ± 0.7 ^a^
MSP3	9.6 ± 3.3 ^d^	7.7 ± 1.3 ^d^	3.7 ± 1.1 ^c^	4.6 ± 0.8 ^c^
MSP4	4.9 ± 1.2 ^c^	2.7 ± 1.2 ^c^	3.4 ± 1.2 ^abc^	2.2 ± 1.1 ^ab^
MSP5	5.7 ± 1.8 ^c^	2.5 ± 1.3 ^c^	4.2 ± 1.5 ^abc^	2.5 ± 0.5 ^b^
MSP6	4.3 ± 0.7 ^bc^	2.4 ± 1.2 ^bc^	3.4 ± 1.4 ^abc^	1.9 ± 0.9 ^ab^

Note: MS, Murashige and Skoog medium. Values are presented as mean ± standard error. Different lowercase letters within each column indicate statistically significant differences among treatments based on Tukey’s post hoc test (*p* < 0.05).

**Table 6:** Effect of different concentrations of growth regulators on the number of de novo microshoot formation from bulb explants in *T. auliekolica* and *T. turgaica*.

Culture Medium	Number of De Novo Microshoot Formation/Explant					
	*T. auliekolica*			*T. turgaica*		
	First Subculture	Second Subculture	Third Subculture	First Subculture	Second Subculture	Third Subculture
MSP1	2.11 ± 0.6 ^abcd^	1.89 ± 0.6 ^abc^	1.0 ± 0.9 ^a^	1.79 ± 0.7 ^abcd^	1.51 ± 0.5 ^abc^	1.22 ± 0.3 ^ab^
MSP2	1.32 ± 0.4 ^ab^	1.13 ± 0.3 ^abc^	1.0 ± 0.4 ^a^	1.51 ± 0.5 ^abc^	1.23 ± 0.4 ^ab^	1.0 ± 0.2 ^ab^
MSP3	4.54 ± 1.4 ^g^	3.97 ± 1.3 ^fg^	3.4 ± 1.1 ^efg^	3.97 ± 1.3 ^f^	3.78 ± 1.2 ^f^	3.02 ± 1.0 ^ef^
MSP4	2.46 ± 0.8 ^bscde^	2.08 ± 0.7 ^abcd^	1.89 ± 0.6 ^abc^	1.98 ±0.5 ^abcde^	1.51 ±0.5 ^abc^	0.95 ± 0.3 ^a^
MSP5	3.54 ± 1.0 ^efg^	3.21 ± 1.0 ^def^	3.02 ± 0.9 ^cdef^	3.02 ± 0.9 ^ef^	2.65 ± 0.8 ^de^	2.46 ± 0.7 ^cde^
MSP6	2.56 ± 0.7 ^cde^	1.98 ± 0.7 ^abcd^	1.8 ± 0.4 ^abc^	2.51 ± 0.5 ^cde^	2.03 ± 0.6 ^bcde^	1.51 ± 0.5 ^abc^

Note: MS, Murashige and Skoog medium. Values are presented as mean ± standard error. Different lowercase letters within each column indicate statistically significant differences among treatments based on Tukey’s post hoc test (*p* < 0.05).

**Table 7:** Effect of different auxin types and concentrations on mature bulb formation in *T. auliekolica* and *T. turgaica*.

Culture Medium		*T. auliekolica*		*T. turgaica*	
	Auxin (mg/L)	Microbulbs per Explant	Bulb Weight (mg)	Microbulbs per Explant	Bulb Weight (mg)
MSB1	IAA 0.5	4.50 ± 1.6 ^ab^	2.15 ± 0.8 ^b^	4.20 ± 1.3 ^ca^	1.52 ± 0.3 ^ca^
MSB2	IAA 1.0	4.47 ± 1.4 ^ab^	1.49 ± 0.6 ^ab^	3.85 ± 1.1 ^bca^	1.45 ± 0.3 ^bca^
MSB3	IAA 2.0	3.46 ± 0.8 ^a^	1.02 ± 0.5 ^a^	1.98 ± 0.7 ^a^	0.88 ± 0.3 ^a^
MSB4	IBA 0.5	7.69 ± 1.4 ^ca^	3.74 ± 0.4 ^ca^	6.18 ± 0.6 ^cb^	2.95 ± 0.3 ^cb^
MSB5	IBA 1.0	5.38 ± 1.5 ^b^	1.90 ± 0.4 ^b^	4.45 ± 0.7 ^ca^	1.06 ± 0.4 ^ab^
MSB6	IBA 2.0	3.80 ± 1.3 ^ab^	1.54 ± 0.3 ^ab^	2.93 ± 0.5 ^ab^	0.85 ± 0.2 ^a^

Note: MS, Murashige and Skoog medium. IAA, indole-3-acetic acid; IBA, indole-3-butyric acid. Values are presented as mean ± standard error. Different lowercase letters within each column indicate statistically significant differences among treatments based on Tukey’s post hoc test (*p* < 0.05).

**Table 8:** Concentrations of different growth regulators in various media for microshoot proliferation of *Tulipa auliekolica* and *T. turgaica*.

Culture Medium	Growth Regulators (mg/L)					
	BAP	TDZ	NAA	IBA	mT	2iP
MSP1	5.0	0.1	0.1			
MSP2	5.0	0.1		0.1		
MSP3	5.0		0.1		0.1	
MSP4	5.0			0.1	0.1	
MSP5	5.0		0.1			0.1
MSP6	5.0			0.1		0.1

BAP, 6-Benzylaminopurine; TDZ, thidiazuron; NAA, 1-naphthylacetic acid; IBA, indole-3-butyric acid; mT, meta-topolin; 2iP, N^6^-(2-isopentenyl) adenine.

## Data Availability

The original contributions presented in the study are included in the article material; further inquiries can be directed to the corresponding author.

## Supplementary Materials

The following supporting information can be downloaded at: https://www.mdpi.com/article/10.3390/plants14193014/s1, Table S1: Habitat characteristics and population density of *Tulipa turgaica* and *T. auliekolica* in Northern Kazakhstan; Table S2: Morphological characteristics of *Tulipa turgaica* and *T. auliekolica* plants; Table S3: ANOVA summary for the effects of temperature, medium type, and their interaction on the germination percentage of *Tulipa auliekolica*; Table S4: ANOVA summary for the effects of temperature, media type, and their interaction on the germination percentage of *Tulipa turgaica*; Table S5: Two-way ANOVA summary for the time to 50% seed germination (T_50_) across temperature, media, and plant species for *Tulipa turgaica* and *T. auliecolica*.

## Abbreviations

The following abbreviations are used in this manuscript:

2iP	N^6^-(2-isopentenyl)adenine
ABA	Abscisic acid
ANOVA	Analysis of variance
BAP	Linear dichroism
GA_3_	Gibberellic acid
HSD	Honestly significant difference
IBA	Indole-3-butyric acid
mT	Meta-topolin
NAA	1-naphthylacetic acid
T_50_	Time to 50% seed germination
TDZ	Thidiazuron
TTC	2,3,5-triphenyltetrazolium chloride
