# Supplementary material for: In Vitro Propagation of Endemic Kazakh Tulips: Effects of Temperature and Growth Regulators
Source: Plants (Basel). 2025 Sep 29;14(19):3014. doi: 10.3390/plants14193014 (PMC12525915; doi:10.3390/plants14193014)
Supplement: Supplementary file 1 [file plants-14-03014-s001.zip › plants-3897634-supplementary.pdf]

Table S1. Habitat characteristics and population density of *Tulipa turgaica* and *T. auliekolica* in Northern Kazakhstan.

Species	Location	Coordinates	Habitat description	Phytocenosis	Number of generative individuals per 1 m^2
T. turgaica	Kostanay region, Amangeldy district, near Tasty village, vicinity of Keiki-Batyr Mausoleum	N 66.026724° E 50.486579°	Flat relief with a shallow depression near a brook; loamy soils. Total population area: 0.3 ha	Grassland-wormwood-dropwort with dominance of *Festuca valesiaca* Gaudin and *Artemisia schrenkiana* Ledeb	10.3 ± 0.6
T. auliekolica	Kostanay region, Aulikol district, 10 km from Karamendy turnoff on Karamendy – Auliekol road	N 64.395259° E 51.983026°	Flat terrain with a gentle south-west slope; loamy chestnut soils. Total population area: 0.1 ha	Couch grass-forb vegetation dominated by *Elytrigia repens* (L.) Nevski	2.0 ± 0.8

Table S2. Morphological characteristics of *Tulipa turgaica* and *T. auliekolica* plants.

Species	Plant height (cm)	Stem diameter (cm)	Leaf blade		Number of flowers per plant	Flower diameter (cm)	Capsule length (cm)	Bulb diameter (cm)	Seed size (mm)	Weight of 1000 seeds (g)
			length (cm)	width (cm)						
T. turgaica	14.43 ± 2.96	0.23 ± 0.05	8.77 ± 2.10	0.51 ± 0.19	1.20 ± 0.41	3.49 ± 0.79	1.35 ± 0.02	1.6 ± 0.50	3.5 x 2.8	6.234
T. auliekolica	10.83 ± 1.67	0.20 ± 0	8.73 ± 1.89	0.43 ± 0.10	1.00 ± 0	2.36 ± 1.13	1.02 ± 0.05	2.0 ± 0.67	5.5 x 4.5	6.484

Table S3. ANOVA summary for the effects of temperature, medium type, and their interaction on the germination percentage of *Tulipa auliekolica.*

**Variable Factor**	Df	**Sum of Squares**	**Mean Square**	**F-value**	***p*-value**
Temperature	4	27406	6852	1454.35	< 2e-16 ***
Medium type	2	1061	530	112.59	1.13e-14 ***
Temperature x Medium	8	487	61	12.91	7.71e-08 ***
Residuals	30	141	5		

Note: Df, degrees of freedom; *** $p < 0.001$; ** $p < 0.01$; * $p < 0.05$

Table S4. ANOVA summary for the effects of temperature, media type, and their interaction on the germination percentage of *Tulipa turgaica.*

**Variable Factor**	**Df**	**Sum of Squares**	**Mean Square**	**F-value**	***p*-value**
Temperature	4	10826	2706.6	322.21	< 2e-16***
Medium	2	5019	2509.5	298.75	< 2e-16***
Temperature x medium	8	3584	448.0	53.33	1.24e-15***
Residuals	30	252	8.4		

Note: Df, degrees of freedom; *** $p < 0.001$; ** $p < 0.01$; * $p < 0.05$

Table S5. Two-way ANOVA summary for the time to 50% seed germination (T_{50}) across temperature, media, and plant species for *Tulipa turgaica* and *T. auliekolica*.

**Variable Factor**	**Df**	**Sum of squares**	**Mean square**	**F-value**	***p*-value**	**Significance**
Temperature	1	45.56	45.56	41.027	<0.001	***
Medium	2	306.46	153.23	137.976	<0.0001	***
Plant	1	1.65	1.65	1.483	>0.05	ns
Temperature × Medium	2	38.58	19.29	17.372	<0.001	***
Temperature × Plant	1	12.6	12.6	11.348	<0.01	**
Medium × Plant	2	3.71	1.85	1.669	>0.05	ns
Temperature × Medium × Plant	2	0.61	0.31	0.275	>0.05	ns
Residuals	24	26.65	1.11			

Note: Df, degrees of freedom; *** $p < 0.001$; ** $p < 0.01$; * $p < 0.05$
